# Structural basis for CD97 recognition of the decay-accelerating factor CD55 suggests mechanosensitive activation of adhesion GPCRs

**DOI:** 10.1016/j.jbc.2021.100776

**Published:** 2021-05-14

**Authors:** Minghui Niu, Shengzhao Xu, Jie Yang, Deqiang Yao, Na Li, Jie Yan, Guisheng Zhong, Gaojie Song

**Affiliations:** 1 Shanghai Key Laboratory of Regulatory Biology, Institute of Biomedical Sciences and School of Life Sciences, East China Normal University, Shanghai, China; 2 iHuman Institute, ShanghaiTech University, Shanghai, China; 3 National Facility for Protein Science in Shanghai, Zhangjiang Lab, Shanghai Advanced Research Institute, CAS, Shanghai, China; 4 Department of Physics, National University of Singapore, Singapore

**Keywords:** adhesion GPCR, CD97, CD55, structure, immune disorder, mechanosensing, tensile force, shearing geometry, 7TM, seven-transmembrane, EGF, epidermal growth factor, EMR, EGF module–containing mucin-like hormone receptor, GAIN, GPCR autoproteolysis-inducing, GPCRs, G protein–coupled receptors, SAXS, small-angle X-ray scattering, SCR, short consensus repeat, SPR, surface plasmon resonance, TEV, tobacco etch virus

## Abstract

The adhesion G protein–coupled receptor CD97 and its ligand complement decay-accelerating factor CD55 are important binding partners in the human immune system. Dysfunction in this binding has been linked to immune disorders such as multiple sclerosis and rheumatoid arthritis, as well as various cancers. Previous literatures have indicated that the CD97 includes 3 to 5 epidermal growth factor (EGF) domains at its N terminus and these EGF domains can bind to the N-terminal short consensus repeat (SCR) domains of CD55. However, the details of this interaction remain elusive, especially why the CD55 binds with the highest affinity to the shortest isoform of CD97 (EGF_1,2,5_). Herein, we designed a chimeric expression construct with the EGF_1,2,5_ domains of CD97 and the SCR_1–4_ domains of CD55 connected by a flexible linker and determined the complex structure by crystallography. Our data reveal that the two proteins adopt an overall antiparallel binding mode involving the SCR_1–3_ domains of CD55 and all three EGF domains of CD97. Mutagenesis data confirmed the importance of EGF_5_ in the interaction and explained the binding specificity between CD55 and CD97. The architecture of CD55–CD97 binding mode together with kinetics suggests a force-resisting shearing stretch geometry when forces applied to the C termini of both proteins in the circulating environment. The potential of the CD55–CD97 complex to withstand tensile force may provide a basis for the mechanosensing mechanism for activation of adhesion G protein–coupled receptors.

Adhesion G protein–coupled receptors (GPCRs) are a subfamily of GPCRs that participate in a wide variety of functions from cell adhesion to immune defense and development, and, consequently, their dysfunction is linked to a myriad of negative health effects including inflammation, neurological disease, and cancer (1). Adhesion GPCRs are characterized by variable tandem adhesion domains followed by a common GPCR autoproteolysis-inducing (GAIN) domain at the extracellular region and a canonical seven-transmembrane (7TM) domain at the C-terminal region (2). The epidermal growth factor (EGF) subgroup of the adhesion GPCR includes five members, CD97, and EGF module–containing mucin-like hormone receptor (EMR) numbers 1 through 4. Each of these members exhibits similar structural patterns but with variant numbers of EGF-like domains. Uniquely among the members, CD97 is widely expressed on granulocytes, monocytes, macrophage, dendritic cells, and smooth muscle cells (3). Recent *in vivo* studies using mAbs have indicated that CD97 plays a prominent role in neutrophil migration and antibacterial immunity (4). Furthermore, CD97 has also been identified as a tumor-associated receptor and it is significantly upregulated in many carcinomas, including gastric, colorectal, and pancreatic (5, 6, 7).

CD97 has three alternative gene-spliced isoforms containing between three and five EGF domains: EGF_1,2,5_, EGF_1,2,3,5_, and EGF_1–5_ (8). These isoforms have been linked to distinct functions, an aspect believed to derive from the different ligands they accommodate (9, 10). CD97 was first reported to bind CD55 (or decay-accelerating factor), a regulator of the complement system (11). Antibody blocking, domain deletion, and swapping experiments have verified the critical role of the first two EGF domains of CD97 in binding CD55 (12, 13). Conversely, the presence of EGF_3–4_ has been shown to reduce CD97 binding affinity with CD55. In addition to CD55, CD97 can also bind to a number of other ligands including CD90 (Thy1) (14) and integrins (15) α5β1 and αvβ3, which likely bind to the GAIN domain, as well as chondroitin sulfate B (16), which binds to the EGF_4_ domain. These varied binding partners, together with the different isoforms of CD97, likely associate with distinct physiological consequences.

CD55 is a GPI-linked membrane protein with four short consensus repeat (SCR) domains at the N terminus. CD55 regulates the complement cascade by inactivating the C3 convertases and plays a critical role in inflammation and pathogen defense (17). The binding of CD55 to CD97 can protect several cell types from complement-mediated damage, and the CD55–CD97 interactions are involved in the pathogenesis for multiple sclerosis (18), synovial inflammation, and rheumatoid arthritis (19). CD55 is also linked to adaptive immunity *via* costimulating CD4^+^ T cells with CD97, resulting in T cell activation and an increase in cell proliferation and cytokine secretion (20).

CD97 shares the highest similarity with EMR2 in the primary sequence, with only three residual differences in EGF domains 1, 2, and 5. However, EMR2 (EGF_1,2,5_) has been found to bind to CD55 with a dissociation constant (*K*_*D*_) much lower (>10 fold) than that of CD97 (EGF_1,2,5_) (13, 21). Crystal structures of the SCR domains of CD55 (22) and the EGF_1,2,5_ domains of EMR2 (23) reveal an extended rod-like conformation. However, owing to the absence of crystal structure for CD55 in complex with CD97 or EMR2, insights into the binding mode and specificity, as well as the signal transduction mediated by the ligand-receptor pair, remain elusive. Herein, we report the structure of the adhesive domains of the CD97–CD55 complex as determined at 3.19 Å resolution by X-ray crystallography. Evaluation of the complex structure reveals not just an overall antiparallel binding mode but also the specificity for CD97 recognition by CD55.

## Results

### Structural determination

**Figure 1:**
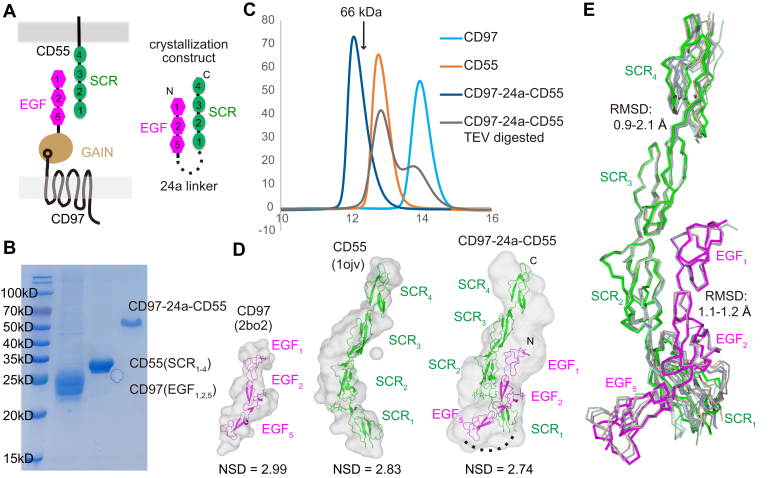
**Structural determination of the CD97–CD55 complex.***A*, the CD97–CD55 binding pattern and our chimeric construct. *B*, SDS-PAGE of individual adhesive domains of CD97 or CD55 or the chimeric CD97–24a–CD55 complex. *C*, size-exclusion profiles of above three proteins as well as the chimeric protein digested by TEV. The elution volume of bovine serum albumin (66 kDa) was indicted as a control. *D*, SAXS analysis of above three proteins and alignments with previously determined crystal structures of EMR2 (2bo2) and CD55 (1ojv) and crystal structure determined in the present study (CD97–24a–CD55 chimeric complex). Normalized structural difference (NSD) was indicated for each sample. *E*, superposition of the CD97–CD55 complex with previous individual monomers. Monomers of CD97 are from PDB IDs 2bo2, 2box, and 2bou. Monomers of CD55 are from PDB IDs 1ojv, 1ojy, and 1ojw. CD97 and CD55 in the complex are colored *pink* and *green*, respectively, while other monomers are in *light colors*. EMR, EGF module–containing mucin-like hormone receptor; SAXS, small-angle X-ray scattering.

The CD55 SCR_1–4_ domains and CD97 EGF_1,2,5_ domains were initially purified independently from HEK293 cells. However, the mixture of the two fragments did not yield a stable complex in gel-filtration chromatography, presumably because of the relatively low affinity between the two proteins. Therefore, we designed a 24-residue linker including a tobacco etch virus protease (TEV) site to connect the C terminus of EGF_1,2,5_ and the N terminus of SCR_1–4_, enlightened by a head-to-tail docking mode based on NMR titration (Experimental procedures, Fig. 1*A*) (13, 23). The chimeric construct was then expressed and purified to homogeneity (Fig. 1, *B* and *C*). Cleavage of the linker with TEV generated a size-exclusion profile of two peaks corresponding to the CD55 and CD97 fragments, suggesting each protein within the chimera construct was well folded individually (Fig. 1*C*). The small-angle X-ray scattering (SAXS) measurements and alignments with models of the chimera complex and each individual protein indicated the formation of a stable CD55–CD97 complex, mediated by the flexible linker (Fig. 1*D*, Fig. S1 and Table S1). We then successfully crystallized the chimeric complex and collected the data to 3.19 Å resolution. We solved the structure with molecular replacement using previous high-resolution structures of CD55 and EMR2 as searching models, and the final structure was refined to R_work_ and R_free_ of 0.26 and 0.30, respectively (Table 1).

**Table 1:** Data collection and refinement statistics

	CD97–CD55 complex
Data collection	
Space group	P2_1_
Cell dimensions	
*a*, *b*, *c* (Å)	51.80, 44.25, 116.53
α, β, γ (°)	90, 98.69, 90
Resolution (Å)^a^	33.23–3.19 (3.27–3.19)
Reflections (total/unique)	33,704/8903
*R*_merge_^b^	0.14 (0.77)
CC1/2^c^	0.98 (0.89)
I/σ(I)	7.3 (2.2)
Completeness (%)	99.2 (98.3)
Redundancy	3.8 (4.0)
Refinement	
*R*_work/_*R*_free_	0.267/0.304
R.m.s.d.	
Bond lengths (Å)	0.004
Bond angles (°)	0.899
Ramachandran (%) plot (%)	93.5/6.5/0
Residue range	A/CD97/25–164; B/CD55/35–284
Carbohydrate residues	4
Metal ion	2 calcium
PDB ID	7DO4

a Values for the highest resolution shells are given in parentheses.

b Rmerge = ∑*hkl*∑_i_|I_i_(*hkl*) − <I(*hkl*)>|/∑*hkl*∑_i_|I_i_(*hkl*), where I_i_ (*hkl*) and <I(*hkl*)> are the i and mean measurement of intensity of reflection *hkl*, respectively.

c CC1/2 = Pearson’s correlation coefficient between average intensities of random half data sets for each unique reflection.

Although most of the residues in the complex can be successfully modeled, the linker region is disordered, consistent with its highly hydrophilic property. Crystal packing revealed two major crystal lattices with packing interfaces of 960 Å^2^ and 748 Å^2^, named lattice A and lattice B, respectively (Fig. S2*A*). Previous binding studies of CD55 and CD97 have emphasized the importance of the N-terminal domains of both proteins in recognition, and our designed 24-residue linker was sufficient to connect the 36 Å distance between the C terminus of EGF_1,2,5_ and the N terminus of SCR_1–4_ in lattice A but not long enough to fill the 106 Å gap in lattice B. Furthermore, lattice A binding mode can be fitted into a SAXS envelop with a normalized structural difference of 2.74; this is relatively better than the fittings of lattice B and the previously proposed model from NMR titration (Fig. S2*B*) (23). In considering of the low-resolution envelope calculated from scattering data, we cannot rule out another possibility of an ensemble fit (24) for the chimeric complex in the solution. Therefore, the surface plasmon resonance (SPR) binding studies have been conducted to further support the lattice A binding mode (see below). All these analyses clearly favor physiological relevance of the binding mode in lattice A over lattice B and we therefore only discuss the lattice A binding mode hereafter.

### Binding mode

The CD55 and CD97 bind roughly antiparallel to each other involving each N-terminal tandem domains (Fig. 1*E*). Structures of CD55 and CD97 in the complex superimposed closely with prior monomeric structures, with a C_α_ r.m.s.d. of 0.9 to 2.1 Å and 1.1 to 1.2, respectively (Fig. 1*E*), suggesting no conformational change is needed during binding. Previous structures of CD55 SCR domains have suggested a constant interface of SCR_2_–SCR_3_ and very little variation in the interfaces of SCR_1_–SCR_2_ and SCR_3_–SCR_4_. In our complex structure, the orientation of SCR_2_–SCR_3_ is invariant and the other interfaces are within similar ranges of variation as the CD55 molecules of previous studies (Fig. 1*E*). Similar case in CD97, only slight variation in the EGF_1_–EGF_2_ or EGF_2_–EGF_5_ interface, is monitored when compared with previous monomeric structures of its homolog protein, EMR2 (Fig. 1*E*). In CD97 or EMR2, EGF_2_ and EGF_5_ each bind a calcium ion in the N-terminal tip, which contributes to rigidity within the interface of EGF_1_–EGF_2_ or EGF_2_–EGF_5_. These comparisons, together with apparent disorder of the 24-residue linker between the two molecules, indicated the binding mode between CD55 and CD97 is not affected by the protein engineering.

The total solvent-accessible surface buried by the EGF_1,2,5_–SCR_1–4_ interactions was 1920 Å^2^, an interface area that is above the average for protein–protein interaction (25). All domains, except SCR_4_, are involved in the CD97–CD55 interaction, and this is the probable reason that SCR_4_ possesses relatively poor electronic density compared with other domains in the complex. Our examination of the SCR_4_ domain revealed that only 20% of its surface area are buried by lattice contact, in contrast to 30% to 39% for the other SCR domains. Our complex structure reveals two N-glycosylation sites in CD97 (Asn-38 and Asn-108) and one N-glycosylation site in CD55 (Asn-95), but none of these carbohydrates are involved in the CD97–CD55 interactions.

**Figure 2:**
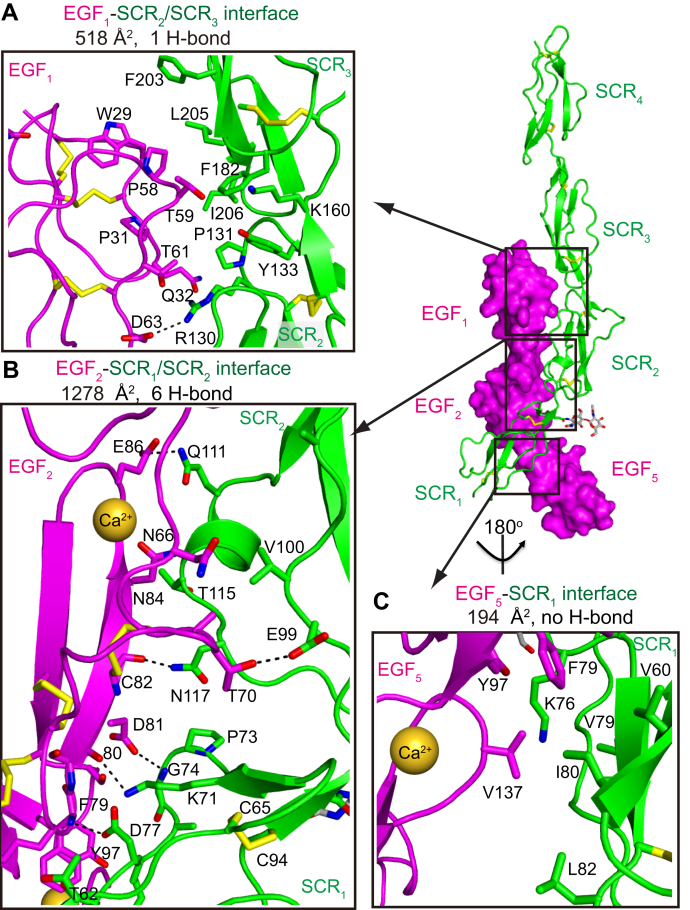
**The detailed interactions of CD97–CD55.***A*–*C*, the detailed interactions of EGF_1_–SCR_2_/SCR_3_ (*A*), EGF_2_–SCR_1_/SCR_2_ (*B*), EGF_5_–SCR_1_ (*C*) interfaces. CD97 and CD55 domains are colored *pink* and *green*, respectively. Interacting residues are shown as *sticks* and labeled. *Dashed lines* indicate hydrogen bonds. *Yellow spheres* represent coordinated calcium. EGF, epidermal growth factor; SCR, short consensus repeat.

The EGF_1,2,5_–SCR_1–4_ interactions can be divided into three interfaces: the EGF_1_–SCR_2_/SCR_3_ interface, the EGF_2_–SCR_1_/SCR_2_ interface, and the EGF_5_–SCR_1_ interface (Fig. 2). In the EGF_1_–SCR_2_/SCR_3_ interface, we observed that Asp-63 of EGF_1_ forms a charged hydrogen bond with Arg-130 of SCR_2_ (Fig. 2*A*). In addition, EGF_1_ also forms extensive hydrophobic interactions with SCR_2_ and SCR_3_ residues and buries a total surface area of 518 Å^2^. The preeminent interactions of CD97–CD55 come from the EGF_2_–SCR_1_/SCR_2_ interface in the middle region, which buries a total surface area of 1278 Å^2^ and contains six pairs of hydrogen bonds (Fig. 2*B*). At the upper half of this interface, Thr-70 and Glu-86 of EGF_2_ hydrogen bond to Glu-99 and Gln-111 of SCR_2_, respectively. In addition, the carbonyl group of Cys-82 in EGF_2_ forms a hydrogen bond to Asn-117 of SCR_2_. At the bottom half of the interface, the EGF_2_–SCR_1_ interactions are secured by three pairs of side chain–main chain hydrogen bonds between EGF_2_ residues Asp-79, Asp-80, and Asp-81 and SCR_1_ residues Asp-77, Lys-71, and Lys-74, respectively. In the third interface of EGF_5_–SCR_1_ (Fig. 2*C*), Val-137 of EGF_5_ inserts into a joint hydrophobic pocket formed by SCR_1_ residues Val-60, Lys-76, Val-79, Ile-80, and Leu-82 and EGF_2_ residue Phe-79. These interfaces are consistent with previous literature, identifying the EGF_1,2_ and SCR_1,2_ domains as critical determinants for CD55–CD97 interactions. Moreover, our structure unveils additional interactions contributed by EGF_5_ of CD97 as well as SCR_3_ of CD55. Notably, the involvement of EGF_5_ in the CD55–CD97 interactions may elucidate why the EGF_1,2,5_ isoform of CD97 shows a higher binding affinity to CD55 than the other two isoforms, EGF_1,2,3,5_ and EGF_1–5_.

**Figure 3:**
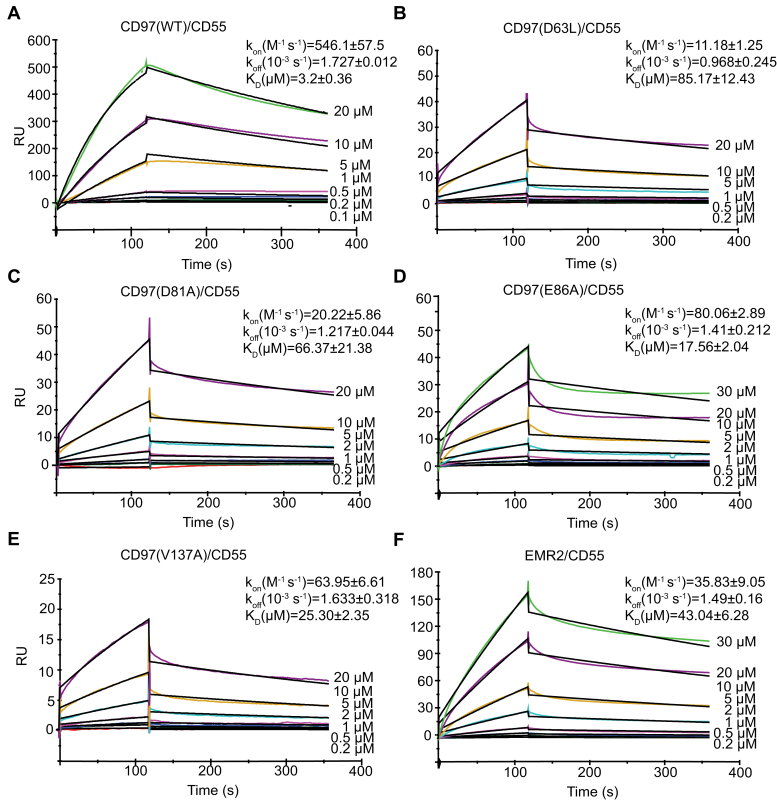
**SPR measurements of WT and mutated CD97 with CD55.** SPR sensorgrams (*colored lines*) are shown with fits (*black lines*). CD55 was immobilized in the NTA chip and titrated with indicated concentrations of CD97 (*A–E*) or EMR2 (*F*). Kinetic values are indicated above the *curves*. Values are the mean ± difference from the mean of two independent experiments. SPR, surface plasmon resonance.

The relatively large interface in the CD55–CD97 complex appears to be inconsistent with the previously characterized low affinity and rapid off-rate binding (13, 25). To understand this puzzle, we remeasured the binding kinetics of CD55 and CD97 by SPR. We preimmobilized His-tagged CD55 onto a nickel coated NTA sensor chip and sequentially injected serial concentrations of CD97 samples. Our results show that CD97 binds to CD55 with a *K*_*D*_ of 3.2 μM, ∼20-fold higher than the previously reported affinity (Fig. 3*A*), and in accord with one study demonstrating a statistical correlation between the interface area and binding affinity (25). Furthermore, compared with the previously determined fast off-rate (13), our measurements show the two proteins bound to each other with a much slower on-rate (546 M^−1^S^−1^) and off-rate (1.73 × 10^−3^S^−1^). These rates are in line with our structural observation of the antiparallel binding interface that composed by tandem rod-like domains at both sides. The apparent difference in binding kinetics between our measurements and previous reports could be attributed to the differing immobilization techniques. In the previous work, CD55 was randomly covalently attached to the chip *via* primary amino groups, which may cause a decrease in flexibility and accessibility by CD97 (13). Moreover, our proteins were both expressed and secreted from HEK293 cells, whereas in the previous work, the CD97 and CD55 proteins were expressed from *Escherichia coli* or *Pichia* (13).

We further measured affinity of CD97 mutants to confirm the physiological relevance of the binding mode. Within the interface, D63, D81, and E86 of CD97 each hydrogen bonds to a residue in CD55 (Fig. 2). Aligned with this interface, the binding affinity of mutants D63L (85.17 ± 2.43 μM), D81A (66.37 ± 21.38 μM), and E86A (17.56 ± 2.04 μM) decreased by 26-, 20-, and 5-fold, respectively (Fig. 3, *B*–*D*).

### Structural specificity for CD97 recognition by CD55

The CD55–CD97 interface defined in our structure does not overlap with the interface proposed from NMR titration (23). In the previous model, the SCR_1_ and N-terminal portion of SCR_2_ in CD55 bind to the other side of CD97 with a much smaller interface (Fig. S3). Furthermore, that model does not explain how such a minute difference between CD97 and EMR2 could dramatically alter the ligand-binding affinity. It also cannot explain why the smallest isoform of CD97 has the highest binding affinity with CD55. In a further demonstration that the previous model is untenable, it locates all three carbohydrates in the binding interface (Fig. S3); the protein samples used for the NMR titration are nonglycosylated as they were generated from *E. coli*. In contrast, our crystal structure reveals key features that illuminate the specificities for CD55 recognition by different splicing isoforms of CD97 as well as its homologues.

Although the interface involved by EGF_5_ (194 Å^2^) is relatively small—and no hydrogen bond is visualized within this interface—this EGF domain may dictate the binding affinity and specificity of the different CD97 isoforms with CD55. In the EGF_1,2,3,5_ and EGF_1–5_ isoforms, the EGF_3_ domain occupies the position of EGF_5_ and faces the SCR_1_ domain of CD55. The key EGF_5_ residue Val-137 is equivalent to a leucine residue in the EGF_3_ domain. The relative conservation of this position suggests that in the other two isoforms, the EGF_3_ domain may involve in the binding of CD55 with similar pattern. To further verify the importance of a hydrophobic aliphatic residue in the interface with CD55, we mutated the V137 to an alanine in the EGF_1,2,5_ fragment and the SPR result showed that the V137A mutant shows an ∼8-fold reduction in binding value (25.3 ± 2.35 μM), confirming the involvement of EGF_5_ in the binding interface with CD55 (Fig. 3*E*). The additional methylene group in the EGF_3_ domain, together with a potential fine-tuning of its orientation compared with the first two EGF domains, may contribute to lower binding affinity of the two longer isoforms (EGF_1,2,3,5_ and EGF_1–5_) than the EGF_1,2,5_ isoform.

**Figure 4:**
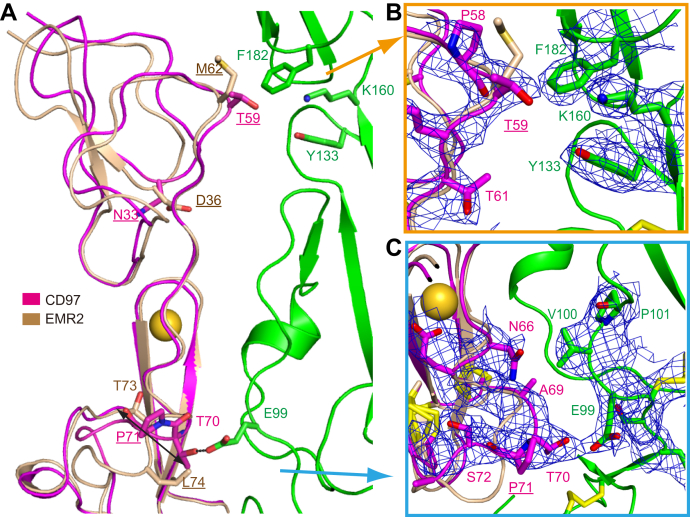
**Comparison of CD97 and EMR2 in their binding with CD55.***A*, for comparison, structure of EMR2 (2bo2) is superimposed to the CD97 EGF domains of the complex. CD97, EMR2, and CD55 are colored *pink*, *dark yellow*, and *green*, respectively. Variant residues are *underlined*, and other key residues are shown as *sticks* and marked. The *double-headed arrow* indicates conformational change of the loop bearing key different residues between CD97 (P71) and EMR2 (L74). *B* and *C*, electronic densities around the variant residues of CD97 and their interacting residues on CD55 are shown at 2Fo − Fc of 1σ. Elements from EMR2 are also shown in panels *B* and *C* for comparison. EGF, epidermal growth factor; EMR, EGF module–containing mucin-like hormone receptor.

The EGF_1,2,5_ fragment of CD97 and EMR2 is differentiated by only three residues, and previous reference suggested >10-fold lower CD55-binding affinity of EMR2 than CD97 (13). Although with different values, our measurements of EMR2 EGF_1,2,5_ fragment indicated similar level of reduction in binding affinity (43.04 ± 6.28 μM), confirming the involvement of these variant residues in the binding interactions (Fig. 3*F*). Two of three variant residues are located in the binding interface with CD55, and their side chains can be unambiguously modeled in the CD97–CD55 complex (Fig. 4). The first variation is located in the EGF_1_–SCR_2_/SCR_3_ interface, where Thr-59 (equivalent to Met-62 in EMR2) of the CD97 EGF_1_ domain encounters the residues Tyr-133, Lys-160, and Phe-182 of CD55. The hydroxyl group of Thr-59 is only ∼4 Å away from Tyr-133 and Lys-160, and this forms a weak polar interaction in the CD97–CD55 interface, whereas similar interaction would be absent in the EMR2–CD55 interface (Fig. 4, *A* and *B*). The second differing residue is located in a thermal-dynamic loop region in each EGF_2_ domain. In CD97, the Pro-71 and its preceding residue Thr-70 adopt a *cis*-peptide conformation, allowing the Thr-70 to flip its backbone and form a hydrogen bond to Glu-99 in the CD55 SCR_2_ domain (Fig. 4, *A* and *C*). A *cis*-peptide conformation is not allowed in EMR2, as the equivalent residue of Pro-71 is Leu-74. Remarkably, in EMR2, the Leu-74 occupies the position of CD97 residue Thr-70, and therefore, no hydrogen bond is formed in this region when it binds to CD55. The third residue difference, Asn-33/Asp-36, is not located in the binding interface, so it may not directly affect the binding of CD97–EMR2 with CD55.

There are conservations, as well as specializations, in the CD97–CD55 interface between different mammalian species. The hydrogen-bonded residues of CD97, Asp-63 and Glu-86, are invariantly Asp/Glu in all species, whereas Asp-81 is mostly conserved but replaced by a Met in rodents (Fig. S4). In CD55, the hydrogen-bonded Asn-117 and Arg-130 are absolutely conserved, and Lys-71 and Asp-77 are highly conserved, except in rodents where they are replaced by Gln and Asn, respectively (Fig. S5). Other hydrogen-bonded residues, including Thr-70 of CD97 and Glu-99 and Gln-111 of CD55, are relatively diverse among species. Remarkably, these unique residues in rodents locate mostly in the EGF_2_–SCR_1_ interface, indicating substantial specialization in the rodent interface. In line with this specialization, previous references have shown that the CD97–CD55 interaction is species restricted and no cross-reaction is found between human and mouse agents (26, 27). Similar to human CD97, mouse CD97 also includes three isoforms but with slightly different organization: EGF_1,2,4_, EGF_1,2,3,4_, and EGF_1,2,X,3,4_ (X refers to a sequence of 45-residues that shows no homolog to any known module) (Fig. S4) (27). It will be interesting to know how the X module may affect the domain orientation and binding specialization with CD55. Despite these specializations, the physiologic function of the CD97–CD55 interaction was proved to be conservative between humans and rodents (19, 28, 29).

### Domain organization of CD97, CD55, and their complex

**Figure 5:**
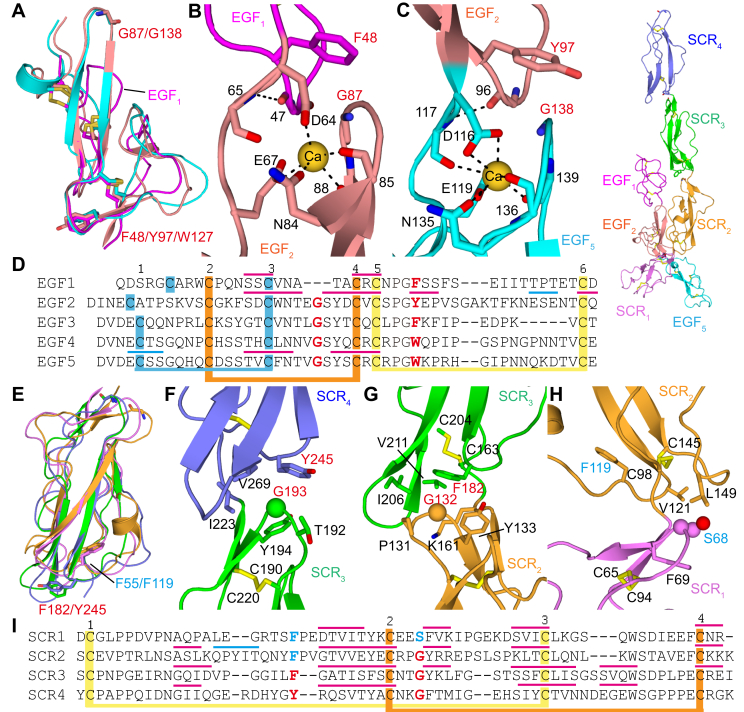
**Sequence analysis of the complemented CD97–CD55 binding mode.***A*, alignment of the three EGF domains within CD97. *B* and *C*, interactions of the interdomain EGF_1_–EGF_2_ and EGF_2_–EGF_5_ interfaces. *D*, sequence alignment of all five EGF domains of CD97. *E*, superposition of SCR domains of CD55. *F*–*H*, CD55 interdomain interfaces. *I*, sequence alignment of all four SCR domains of CD55. Color codes are shown at the *top right*. Interacting residues are shown as *sticks* and labeled, while in panels *F*–*H*, the key G193/G132/S68 residues are shown as *spheres*. In panels *D* and *I*, *thick lines* indicate disulfide bonds, while *cyan* and *red thin lines* indicate α-helices and β-strands, respectively. In panels *A*–*D*, the conserved aromatic residues and glycine residues are marked *red*, while in panels *E*–*I*, the structurally unconserved aromatic residues F55/F119 or hydrophilic S68 are marked *cyan*. EGF, epidermal growth factor; SCR, short consensus repeat.

Within the three structured EGF domains of CD97, EGF_1_ is immediately different from the other two domains because of its shorter β-sheet (Fig. 5, *A* and *D*). The EGF_1_–EGF_2_ and EGF_2_–EGF_5_ interfaces are almost identical, featuring a main chain–main chain hydrogen bond and a van de Waals interaction between a glycine residue (Gly-87/Gly-138) in the β-turn of the EGF_2_ or EGF_5_ domain and a conserved aromatic residue (Phe-48/Tyr-97) in the precedent domain (Fig. 5, *B*–*D*). The aromatic and the non–side-chain glycine residues are also conserved in the EGF_3_ and EGF_4_ domains (Fig. 5*D*), indicating similar domain orientations in the EGF_1,2,3,5_ and EGF_1–5_ isoforms. CD55 also contains similar van de Waals interactions between the preceding SCR_2_ (Gly-132) and SCR_3_ (Gly-193) domains and their following SCR_3_ (Phe-182) and SCR_4_ (Tyr-245) domains (Fig. 5, *E*–*G*), respectively. Moreover, the van de Waals interactions in the SCR_2_–SCR_3_ and SCR_3_–SCR_4_ interfaces are each surrounded by additional hydrophobic residues that strengthen each interface. Nevertheless, in the SCR_1_ and SCR_2_ domains, the corresponding aromatic residues (Phe-55/Phe-119) are flipped and pointed toward the hydrophobic core of each domain (Fig. 5, *E*–*H*). Furthermore, the glycine residue is not conserved in SCR_1_ and its replacement by a serine residue generates a perturbation in the hydrophobic cage, making an orientation similar to SCR_2_/SCR_3_ or SCR_3_/SCR_4_ energetically unfavorable (Fig. 5, *H* and *I*). To expose the hydrophilic Ser-68, the SCR_1_ is tilted about 45° to make contact with SCR_2_; this is in contrast to roughly straight orientations between the other SCR domains. The SCR_1_–SCR_2_ contact buries a total surface area of 560 Å^2^, the largest among all interdomain contacts in CD55 or CD97.

Together with SCR_2_, the tilted SCR_1_ appears like a hook that holds the body of the CD97 EGF_2_ domain. The majority of the interactions of CD55 with CD97 come from this SCR_1_–SCR_2_ pair, including six of the seven total hydrogen bonds between the two proteins. One example is the Thr-70 bearing loop of CD97 EGF_2_ domain, which reaches deep into the hook and hydrogen bonds to the Glu-99 of CD55 (Fig. 2). In addition, CD55 has a unique α-helix in the SCR_2_ domain that interacts and complements the first strand of EGF_2_ in CD97 (Fig. 5*I*). Adjacent to the hook is a conserved SCR_2_/SCR_3_ pair that generates a mostly hydrophobic patch engaging the EGF_1_ domain. Such domain pairs playing important roles for ligand binding are not uncommon and have been observed in the case of CD46 (30, 31). Both CD55 and CD97 contain 3 to 5 residues in the interdomain linkers, and these shorter linkers enable numerous interdomain contacts and constant domain orientations. These sequence and structural features elucidate the unique orientations of both the EGF and SCR domains, which facilitate an elongated and complementary interface between CD55 and CD97.

## Discussion

The CD55–CD97 pair has been indicated to play an important role in host defense and inflammation, as they can mediate cell adhesion and prevent the uncontrolled clustering of leukocytes in the blood stream (20, 32). Specifically, during inflammation conditions, leukocytes bearing CD97 are targeted toward and adhere to the inflammation site through multiple contacts including the CD55–CD97 pair. Despite a slow on-rate, CD97 is in close proximate distance to CD55 during cell adhesion and clustering; thereby, fast formation of a linkage between these two molecules is feasible as a result of high local concentration. The overall architecture of the CD55–CD97 binding mode together with structural details leads to a shearing stretch geometry, which is known to resist force applied to the protein interface (33). For example, the first strand of EGF_2_ forms several hydrogen bonds with CD55; this strand adopts the most force-resistant orientation, which is parallel to the force vector through the C termini of CD55 and CD97, thus can hardly be peeled off. It is therefore reasonable to assume that the slow off-rate of the CD55–CD97 complex (1.73 × 10^−3^s^−1^ measured by SPR) is retained over a significant force range. The potential capability of withstanding a significant range of mechanical force suggests that the CD55–CD97 may mediate force transmission to the following GAIN domain, which is believed to be important for the function of adhesion GPCRs. For many adhesion GPCRs, the 7TM domain is noncovalently associated with the extracellular region through its very N-terminal fragment (also called *Stachel* sequence, or tethered agonist) that inserts into a β-sheet module of the extracellular GAIN domain (2, 34). The transmitted shear force may induce a conformational change of the GAIN domain and separate it from the *Stachel* sequence (Fig. S6). Therefore, when leukocytes become overclustered, CD97 could be downregulated upon mechanically releasing its extracellular region from the cell membrane. Through this mechanism, uncontrolled clustering of leukocytes can be avoided in the circulation (32). Importantly, the mechanically exposed *Stachel* sequence has been testified to reorient and bind the 7TM domain, triggering downstream signaling pathways (35, 36, 37, 38). Recently, another SCR-containing complement regulator, factor H–related protein 1 (FHR1), was reported to bind EMR2 and trigger the downstream phospholipase C pathway (39). Although an unambiguous downstream adaptor for CD97 has yet to be revealed, its antiparallel binding modality with CD55 may already provide a template for potential transmission of the tensile force to the GAIN and 7TM domains for downstream signaling (Fig. S6). Nonetheless, mechanical force was reported to induce phosphorylation in the CD97 intracellular PDZ-binding motif, thus triggering a G protein–independent pathway (40).

The concept of mechanosensing mechanism for adhesion GPCRs has been proposed previously (41, 42, 43, 44); the present study is in favor of that force-induced signaling upon revealing an empirical force-resisting ligand-binding geometry. Given the relevance of the CD55–CD97 complex in immune disorders and carcinomas, elucidating this unique complex structure may provide insights for pharmaceutical development and a greater understanding of the mechanisms of the human body’s signaling pathways.

## Experimental procedures

### Protein production and purification

The CD97 (UniProt: P48960) EGF domains, CD55 (UniProt: P08174) SCR domains, and chimeric complex were expressed and purified as previously described (45). Briefly, the codon-optimized EGF_1,2,5_ isoform of CD97 (21–165), CD55 (35–285), or chimeric complex gene was inserted into a customed pLEXm (46) vector with N-terminal signal peptide and C-terminal His_6_ tags. In the chimeric construct (named CD97–24a–CD55), a 24-residue linker (GSGENLYFQSGSSSSGWRGGHVGS) was added to link the C terminus of EGF_5_ and the N terminus of SCR_1_. HEK293S GnTI^−^ were cultured to 1 to 2 million/ml in suspension and were transiently transfected with DNA: polyethylenimine at 1:3 wt/wt. Culture supernatants were harvested after 3 to 4 days. Purification was with nickel coated NTA affinity followed by Superdex S200 increase gel filtration in 20 mM Tris, pH 7.5, 200 mM NaCl, and 2 mM CaCl_2_. The three purified proteins were subjected to SAXS analysis, and chimeric complex was concentrated to 16 mg/ml for crystallization trials. In the SPR measurements, the EGF_1,2,5_ (24–168) fragment of EMR2 (UniProt: Q9UHX3) and the EGF_1,2,5_ fragment of CD97 were cloned to the same vector as above except a TEV protease site was inserted after each gene sequence to remove the C-terminal His_6_ tag during purification. Single point-mutation fragments of CD97 were made by overlapping PCR and cloned to the same vector for expression and purification.

### SAXS

SAXS experiments were performed at beamline BL19U2 of the National Facility for Protein Science Shanghai (NFPS) at Shanghai Synchrotron Radiation Facility. The wavelength (λ) of X-ray radiation was set as 0.918 Å. Scattered X-ray intensities were collected using a Pilatus 1M detector (Dectris Ltd). The sample-to-detector distance was set such that the detecting range of momentum transfer [*q* = 4π sinθ/λ, where 2θ is the scattering angle] of SAXS experiments was 0.008 to 0.47 Å^−1^. To reduce the radiation damage, a flow cell made of a cylindrical quartz capillary with a diameter of 1.5 mm and a wall of 10 μm was used. SAXS data were collected as 20 × 1 s exposures, and scattering profiles for the 20 passes were compared at 10 °C using 60 μl sample in 20 mM Tris, pH 7.5, 200 mM NaCl, and 2 mM CaCl_2_. Measurements were carried out at two different concentrations in all cases using concentrations between 0.5 and 2 mg/ml. The 2D scattering images were converted to 1D SAXS curves through azimuthally averaging after solid angle correction and then normalizing with the intensity of the transmitted x-ray beam, using the software package BioXTAS RAW (47). The scattering data were binned over an interval of 7 pixel data points, and the background scattering was subtracted using PRIMUS in ATSAS software package (48). Pair distance distribution functions of the particles *P(r)* and the maximum sizes D_max_ were computed using GNOM (49). The *ab initio* shapes were determined using GASBOR (50). SAXS data collection, analysis, and modeling fitting are summarized in Table S1.

### Crystallization and structure determination

The concentrated CD97–24a–CD55 complex was set up for crystallization using hanging drop with NT8 (Formulatrix). Diffraction-quality crystals were produced at 18 °C in 0.1 M MES, pH 6.5, and 12% to 15% w/v PEG 20000. Another linker with six repeats of GSGP (GSGPGSGPGSGPGSGPGSGPGSGP) also yielded thin crystals in similar conditions, but we happened to optimize better crystals from the CD97–24a–CD55 construct. In contrast, a shorter linker with 18 residues (GSGGSGGSGGSGGSGGSG) did not crystallize in the same condition. Single crystals were directly frozen in liquid nitrogen. Diffraction data were collected at a wavelength of 0.979 Å at beamline BL17U1 at the Shanghai Synchrotron Radiation Facility and indexed, integrated, and scaled using the automatic XIA2 software package (51). The structure was solved by the molecular replacement method using structures of CD55 (1ojv) and EMR2 (2bo2) as the search model simultaneously. Refinement was carried out using Phenix (52) and with manual adjustments with Coot (53). Refinement parameters are summarized in Table 1.

### SPR measurement

For SPR experiments using Biacore T200 (GE Healthcare), His-tagged CD55 was immobilized on a nickel coated NTA chips. WT and mutant CD97 (without His-tag) were gel-filtered using Superdex 75 increase to remove aggregates before use. Protein was injected at 50 μl/min for 2 min in a buffer containing 20 mM Tris, pH 7.5, 200 mM NaCl, 2 mM CaCl_2_, and 0.05% Tween 20. The surface was regenerated with 4 M MgCl_2_ for 4 min at the end of each cycle to restore resource units to baseline. All traces were corrected for refractive index changes by subtraction of a control trace simultaneously recorded from a mock-immobilized channel. Kinetics and affinity analysis were performed with SPR evaluation software (GE Healthcare).

## Data availability

Atomic coordinates and structure factors for the CD97–CD55 structure have been deposited in the Protein Data Bank with identification code 7DO4. Correspondence and requests for materials should be addressed to gjsong@bio.ecnu.edu.cn (Gaojie Song).

## Supporting information

This article contains supporting information (23).

## Conflict of interests

The authors declare that they have no conflicts of interest with the contents of this article.
