# Supplementary material for: Structural basis for CD97 recognition of the decay-accelerating factor CD55 suggests mechanosensitive activation of adhesion GPCRs
Source: J Biol Chem. 2021 May 14;296:100776. doi: 10.1016/j.jbc.2021.100776 (PMC8191316; doi:10.1016/j.jbc.2021.100776)
Supplement: Supplemental Figures S1–S6 and Tables S1 [file mmc1.pdf]

Table S1. SAXS data collection, analysis, and modeling fitting.

**(a) Sample details**

| | CD97 | CD55 | chimeric complex |
|---|---|---|---|
| Organism | | Homo sapiens | |
| Source (Catalogue No. or reference) | | HEK293S GnTI- expressed | |
| UniProt sequence ID (residues in construct) | P48960(21-165) | P08174(35-285) | CD97-24a-CD55 |
| Extinction coefficient ε (A280, $M^{-1} cm^{-1}$) | 22095 | 39390 | 68475 |
| Partial specific volume ($cm^3$ $g^{-1}$) | 0.715 | 0.718 | 0.723 |
| Mean solute and solvent scattering length densities | 12.361/9.489 | 12.365/9.489 | 12.528/9.489 |
| mean scattering contrast Δρ ($cm^{-2}$) | 2.872 | 2.876 | 3.039 |
| Molecular mass M from chemical composition (KDa) | 16.6 | 28.6 | 46.8 |
| Concentration (range/values) measured and method | 0.5-2 | 0.5-2 | 0.5-2 |
| Solvent composition and source | | 20 mM Tris 7.5,200 mM NaCl and 2 mM $CaCl_2$ | |

**(b) SAS data collection parameters**

| | |
|---|---|
| Source, instrument and description or reference | Ref. J. Appl. Cryst. (2016) 49, p1428-1432 |
| Wavelength (Å) | 0.9184 |
| Beam geometry (size, sample-to-detector distance) | 340μm x 60μm (H x V), 2.415m |
| q-measurement range (Å-1 or nm-1) | 0.008-0.47 |
| Basis for normalization to constant counts | Take silver behenate as standard to set the mask, then normalize 2D images |
| Method for monitoring radiation damage, X-ray dose dose where relevant | SAXS data were collected as continuous serial exposures and scattering profiles for the passes were compared to monitor the radiation damage |
| Exposure time, number of exposures | 1 s per frame, total 20 frames |
| Sample temperature (ºC) | 10 |

**(c) Software employed for SAS data reduction, analysis and interpretation**

| | |
|---|---|
| SAS data reduction | *In I(q)* versus *q* using *OriginPro 8* (http://www.OriginLab.com), solvent substraction using *PRIMUS* (*ATSAS 2.8.0;* Franke *et al*., 2017) |
| Extinction coefficient estimate | http://protcalc.sourceforge.net/ |
| Calculation of Δρ and ΔV values from chemical compo | MULChe ref.Whitten et al., 2008 |
| Basic analyses: Guinier, P(r), Vp | *PRIMUS* (*ATSAS 2.8.0*; Franke et al., 2017) |
| *volume (e.g. Porod volume VP or volume of correlatioı* | PRIMUS (ATSAS 2.8.0; Franke et al., 2017) |
| Shape/bead modelling | *GASBOR*(Svergun *et al.*, 2001) and *DAMAVER* (Volkov *et al*., 2003) |
| Molecular graphics | *PyMOL* |

**(d) Structural parameters**

| | CD97 | CD55 | chimeric complex |
|---|---|---|---|
| Guinier Analysis | | | |
| *I*(0) (a.u) | 11.82±0.32 | 16.27±0.23 | 15.11±0.21 |
| Rg (Å) | 28.27 ± 0.34 | 41.60 ± 0.56 | 33.56 ± 0.39 |
| q-range ($Å^{-1}$) | 0.0255-0.0456 | 0.0187-0.03121 | 0.0259-0.0384 |
| Quality-of-fit parameter (with definition) | 0.71 | 0.79 | 0.88 |
| P(r) analysis | | | |
| I(0) (a.u) | 12.47 | 16.34 | 17.38 |
| Rg (Å) | 31.07 | 43.75 | 41.43 |
| dmax (Å) | 99.5 | 142.5 | 142.5 |
| q-range ($Å^{-1}$) | 0.0255-0.0456 | 0.0187-0.03121 | 0.0259-0.0384 |
| Quality-of-fit parameter (with definition) | 0.753 | 0.737 | 0.792 |
| Volume (e.g. VP and/or Vc) | 39190 | 36290 | 72750 |

**(e) Shape modelling results (a complete panel for each method)**

| | CD97 | CD55 | chimeric complex |
|---|---|---|---|
| q-range for fitting (Å$^{-1}$) | 0.0080-0.4068 | 0.0080-0.4068 | 0.0080-0.4068 |
| Symmetry/anisotropy assumptions | P1 | P1 | P1 |
| χ^2 value/range | 3.09 | 5.27 | 2.23 |
| Model volume and/or *M* estimate | 41742 | 41878 | 50600 |
| Model precision/resolution | 2.394 | 1.982 | 1.982 |

**(f) Atomistic modelling**

| | CD97 | CD55 | chimeric complex |
|---|---|---|---|
| Method | PyMol | PyMol | PyMol |
| q-range for fitting | 0.0138-0.4049 | 0.0138-0.4049 | 0.0134-0.4041 |
| Symmetry assumptions | P1 | P1 | P1 |
| Any measures of model precision | NSD | NSD | NSD |
| NSD value, any other quality-of-fit parameters | 2.99 | 2.83 | 2.74 |

**(g) Data and model deposition IDs**

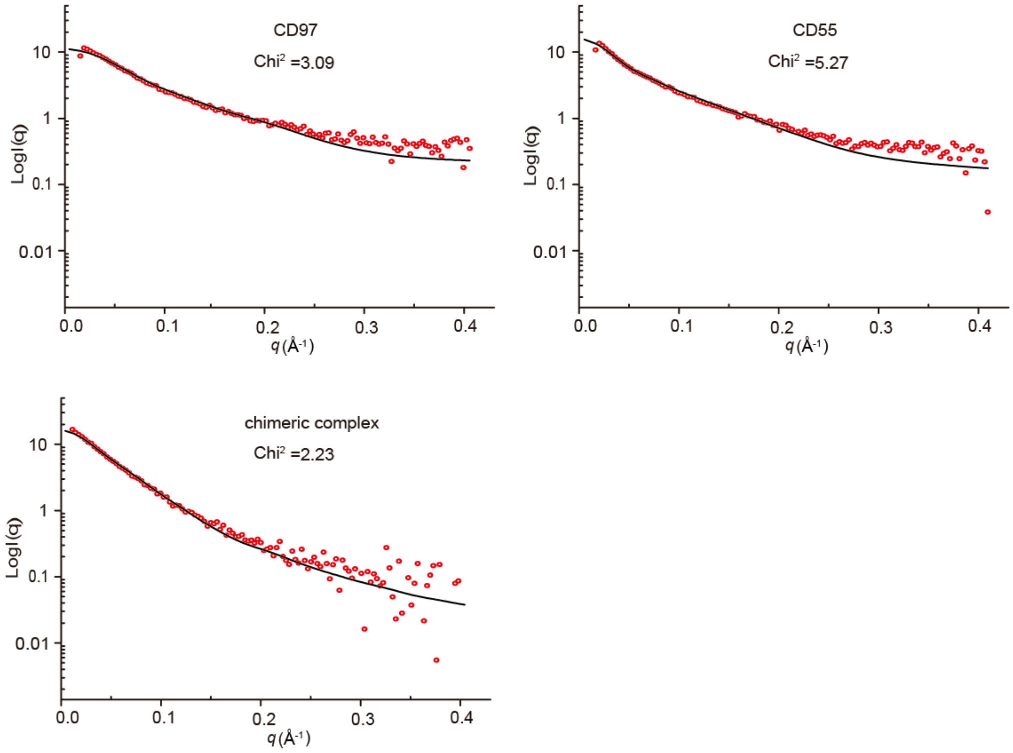


Fig. S1. Raw SAXS data (red points) and fits (black lines) of the CD97–CD55 chimeric complex and individual proteins.

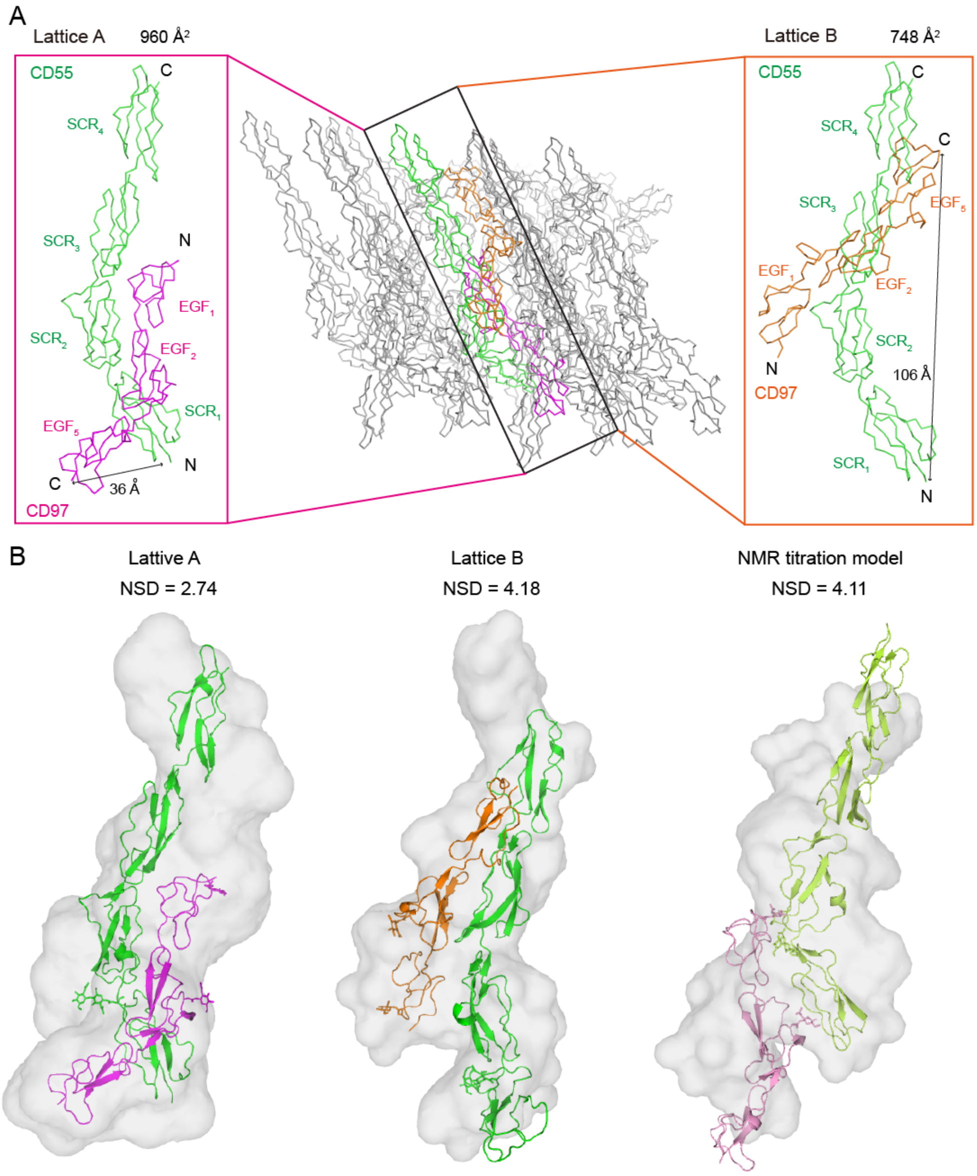


Fig. S2. The packing interfaces and alignments with SAXS envelop of the CD97–CD55 complex. (A) Two major lattices are shown in the pink and orange frames, respectively. Lattice A indicates anti-parallel interaction involving the N-terminal domains of each individual protein; whereas the Lattice B shows a parallel interaction composing each C-terminal domains, which is contradictory with a canonical trans-type ligand-receptor interaction and several recent studies. (B) The two lattices and previous NMR titration model are superimposed with the calculated SAXS envelop of the complex. Lattices A and B are colored according to panel A, while the CD97 and CD55 in the NMR titration model are colored light red and light green, respectively. Smaller NSD (normalized structural difference) indicates better alignment.

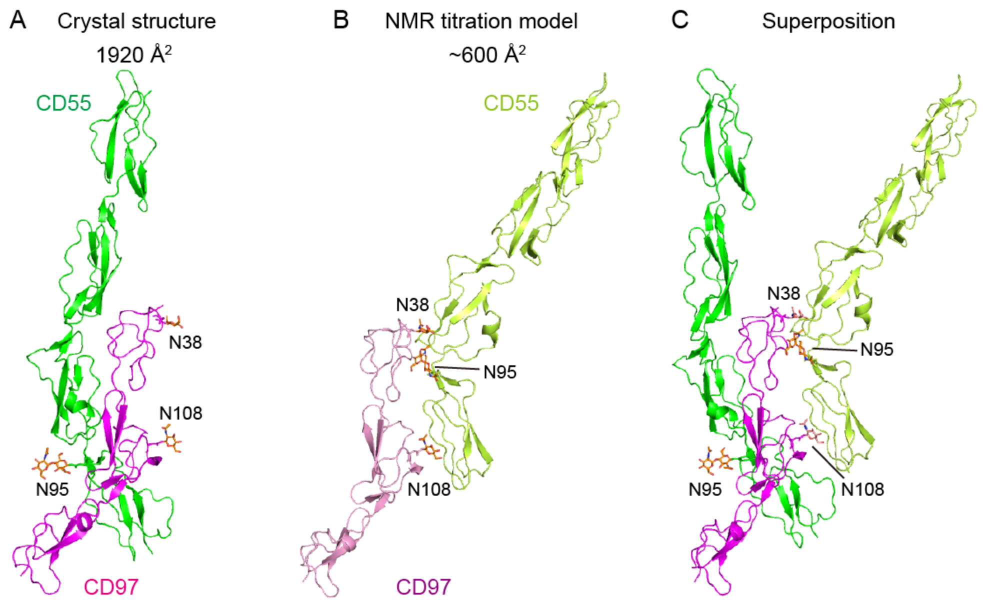


Fig. S3. Comparison of the crystal structure with previous NMR titration model.

The crystal structure (A) and NMR titration model (B) are shown with same orientation on CD97. The NMR titration model is prepared based on figures from previous paper[(23)]. N-glycosylation sites are shown as sticks and marked. C. The two models are superimposed on CD97.

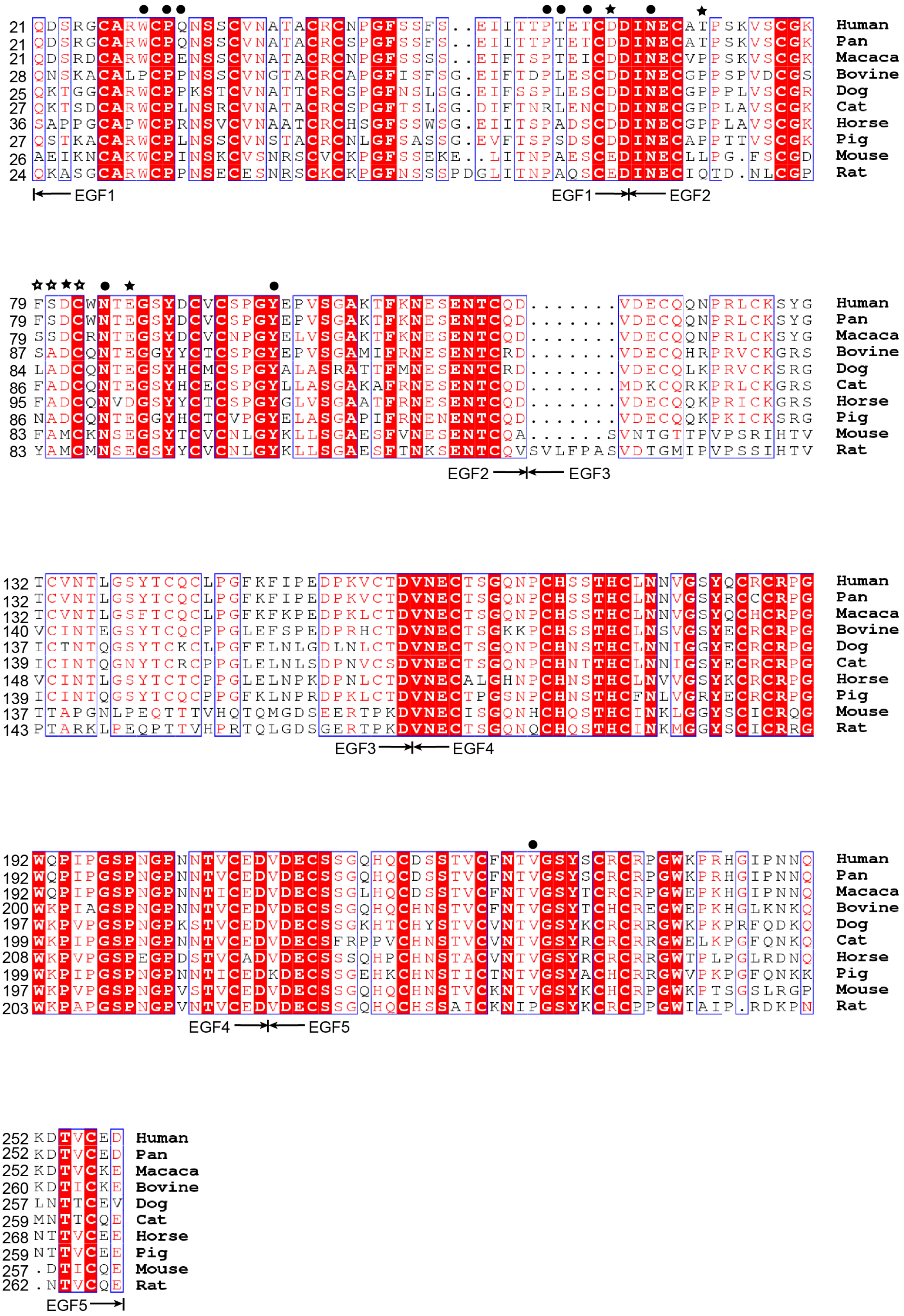


Fig. S4. Sequence alignment of CD97 EGF domains.All five EGF domains of different species were aligned with each EGF domain boundary marked. Each rodent (rat/mouse) CD97 contains a sequence of ~45-residues in the middle and their EGF3 and EGF4 align well with others' 4th and 5th EGF domains. CD55-interacting residues are marked with solid dots, while hydrogen-bonded residues are marked with solid (side chain) and empty (main chain) asterisks.

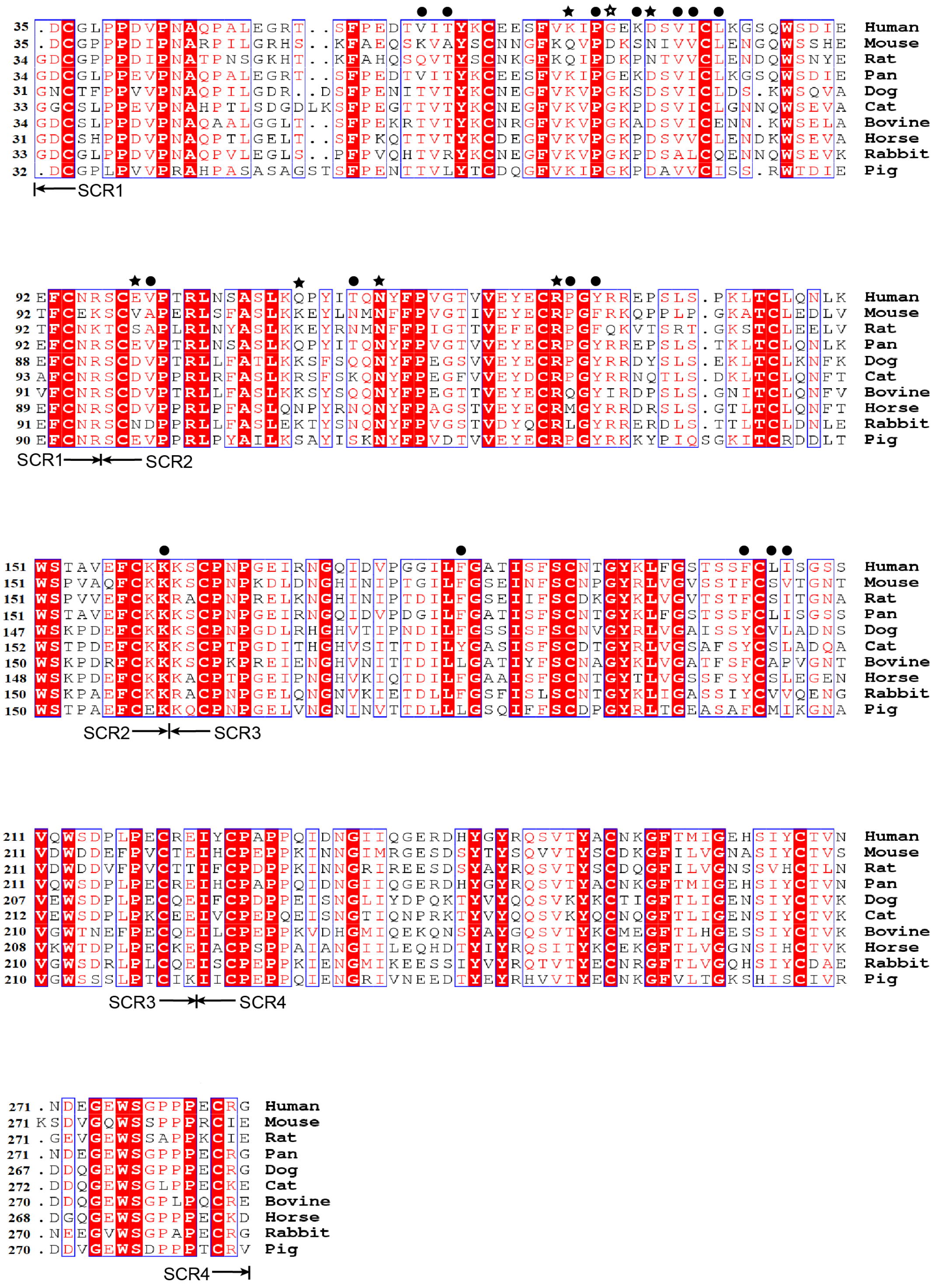

Fig. S5. Sequence alignment of CD55 SCR domains.

Domain boundaries were marked and CD97-interacting residues were marked as supplementary figure 4.

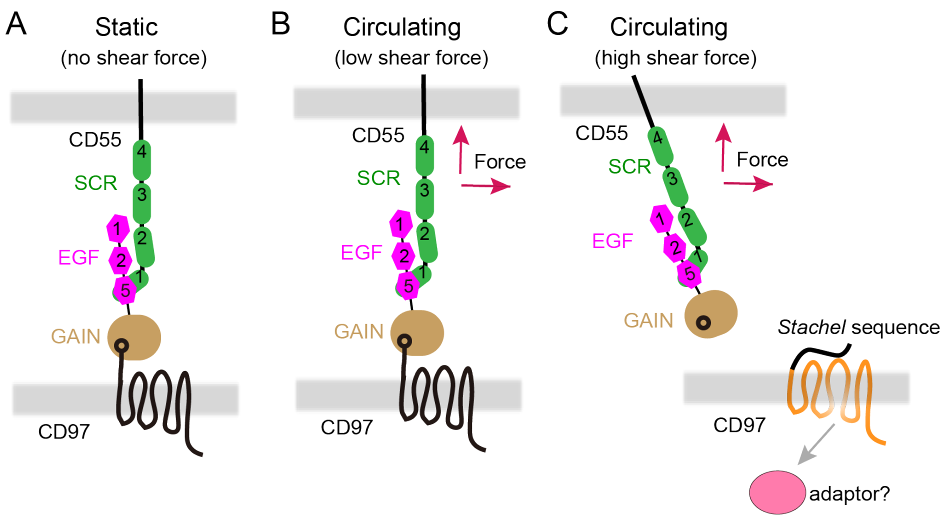


Fig. S6. The CD97–CD55 interaction and signaling.

In static (A) or low shear force (B) tissues, contacts between receptor CD97 and one of its ligands CD55 can withstand a significant range of shear force thus facilitate cell adhesion. The binding modality of CD97 and CD55 suggests a shearing stretch geometry, and indicates transmission of force from the binding interface to the following GAIN and 7TM domains. When the shear force is large enough (C), it can induce conformational change of the GAIN domain and release of the *Stachel* sequence. For example, in inflammation site, certain population of the extracellular region was stretched away by CD55 and dissociated from the cell membrane, thus the leukocytes will not be over clustered. The exposed *Stachel* sequence may reorient and dock to the 7TM domain and trigger downstream signaling, although a downstream adaptor has yet to be identified for CD97.
